# Supplementary material for: Circulating Blood Prognostic Biomarker Signatures for Hemorrhagic Cerebral Cavernous Malformations (CCMs)
Source: Int J Mol Sci. 2024 Apr 26;25(9):4740. doi: 10.3390/ijms25094740 (PMC11084792; doi:10.3390/ijms25094740)

Blood prognostic biomarker signatures for hemorrhagic cerebral cavernous malformations (CCMs)

Jacob Croft[1]*, Brian Grajeda[2]*, Luis A Aguirre[2], Johnathan S Abou-Fadel[1],
Cameron C Ellis[2], Estevao Igor[2], Igor C. Almeida[2], Jun Zhang[1,++]

Department of Molecular & Translational Medicine (MTM)[1]
Texas Tech University Health Science Center El Paso (TTUHSCEP), El Paso, TX 79905 USA
Department of Biological Sciences[2], University of Texas at El Paso, El Paso, TX 79902 USA

Supplemental Materials

***Shared co-first authorship**: Authors who have worked together and contributed equally to this project.

[++]All correspondence:
Jun Zhang, Sc.D., Ph.D.
Department of Molecular and Translational Medicine (MTM)
Texas Tech University Health Science Center El Paso
5001 El Paso Drive, El Paso, El Paso, TX 79905
Tel: (915) 215-4197, Email: jun.zhang2000@gmail.com

Supplemental Legends

Figure S1. A biological function analysis on the number of expressed proteins by CCM1 subjects compared to normal controls using group gene ontogeny (GO).

A-B. Group Gene Ontology (GO) analysis and pathway analysis were performed for biological profiling and systematic functional annotation of the identified group of DEPs in order to determine the statistical significance of a biological process. These key processes indicate the presence of proteins, but their effect on the study subject in both human (**A**) and mouse (**B**) cannot be determined solely based on this information. Supplemental figure 2 provides similar trends in molecular function (Suppl. Fig. 2A, 2C) and cellular processes (Suppl. Fig. 2B, 2D).

C-D. To confirm whether our identified DEPs are frequently associated with specific biological functions, GO enrichment analysis was performed on three distinct subtypes of GO: biological process, molecular function, and cellular component. The molecular function and cellular component subtypes can be observed in supplemental figure 2. It's important to note that these key nodes or processes indicate the presence of proteins, but their positive or negative effect on both human (**C**) and mouse (**D**) study subjects cannot be determined solely based on this information. **E-F.** Volcano plots exhibit differentially expressed proteins (DEPs) between CCM1-deficient subjects and healthy controls. This visualization illustrates proteins present in both control and confirmed CCM-deficient subjects, showcasing differentiations in protein expression in subject with deficient CCM1 compared to normal controls. A p-value, computed from a Student's t-test, is displayed using a -log10 transformation relative to the log2 fold change. This method helps to regulate infinite fold values for unique observations, thereby preventing distortion in the graph. The labeled data points within the plot represent fold changes in protein expressions for CCM1-deficient subjects compared to the control group in both humans (E) and mice (F). The significance of up- and down-regulated proteins from figures E and F was refined using a -log10 p-value threshold to

identify more significant findings.

Figure S2. Group Gene Ontology (GO) analysis on the molecular function and cellular component of the expressed proteins in both human and mouse models with CCM1 deficiency, compared to their respective healthy controls.

A. The group analysis of molecular function in the human CCM1 subject indicated that the majority of the expressed proteins were involved in binding processes. However, it's important to note that the number of proteins measured does not reflect whether these processes are positively or negatively regulated. Nonetheless, the higher number of proteins may suggest an increased demand due to decreased function observed in CCM1 diagnosis. **B.** The cellular component analysis of the human CCM1 subject revealed that the majority of the expressed proteins are involved in cellular anatomical entity. It's important to note that the number of proteins produced does not indicate whether their effects are positive or negative. However, we can speculate that the increased demand for these proteins may be a result of the observed changes in CCM1 diagnosis. **C.** Similar to the human CCM1 subject (Supp. Fig. 2A), the molecular function group analysis of the mouse CCM1 subject showed that the largest number of expressed proteins were involved in binding actions. However, it's important to note that these numbers do not reflect whether the effects of these proteins are positive or negative. Nonetheless, the increased demand for these proteins could be due to the negative effects observed in CCM1 diagnosis. **D.** Similarly, the cellular component group analysis of the mouse CCM1 subject showed that, like the affected human (Supp. Fig. 2B), most of the expressed proteins were involved in the cellular anatomical entity. **E.** This panel depicts the pathway of protein activity within the molecular function pathways of a human CCM1 patient, with highlighted nodes indicating where the measured proteins from the

collected samples interact with the pathway. It provides an expanded view ofhow the binding processes from Supp. Fig. 2A are affected. **F.** This panel illustrates the pathway of protein activity within the cellular components of cells in a human CCM1 subject, highlighting the affected areas that support the CCM1 diagnosis, as well as where the abundant proteins from Supp. Fig. 2B are affecting the cellular components. **G.** This molecular function pathway for the CCM1 mouse illustrates the areas where the harvested proteins affect the pathway, similar to what was observed in the human subject. The nodes represent the different stages of protein activity. As seen in both the mouse and human subjects, the binding activity is where these proteins are predominantly represented. Impaired binding processes are indicative of the CCM1 diagnosis, thus supporting the diagnosis in both laboratory and human subjects through replication. **H.** This cellular component pathway for the CCM1 mouse subject illustrates the areas where the harvested proteins affect the cellular components. These areas, if negatively affected, would support the diagnosis of CCM1, as they are the impaired areas anticipated by the cellular malformations observed in CCM1 diagnosis. The nodes represent different stages of protein activity within the pathway.

Figure S3. GSEA analysis demonstrates the pathways affected in both human and mouse CCM1 subjects.

A. The gene set enrichment analysis upset plots illustrate the pathways that are affected in the CCM1 human subject. The affected pathways support the diagnosis of CCM1 subjects by showing negative regulation of organismal processes, negative responses to binding of cells as observed in wound healing, and negative effects on the organization of cellular processes. This is

consistent with the biological process group GO as shown in supplemental figures 1A. **B**. This gene set enrichment analysis upset plot displays the pathways that are affected in the mouse CCM1 subject. Similar to the human subject, there is a decreased function of the membrane, which supports the diagnosis of CCM1 as the malformation of the plasma membrane results in oblong formations observed in this diagnosis.

Figure S4. GSEA analysis identified enriched pathways for the DEPs in the CCM1-deficiencys compared to their normal conditions, for both human and mouse subjects.

A. The pathways identified in the human CCM1 subjects were compared to age and gender-matched healthy controls, revealing negative regulation of all enriched pathways that are known to be affected by CCM1 deficiency. This supports the previous upset plots that displayed the negatively affected pathways, as well as providing additional insight into the results from the group GO molecular function and cellular components observed in Supp. Fig. S1A-B. **B**. The pathways identified through GSEA analysis of CCM1 mouse compared to a healthy control showed negative effects that signify the affected processes and further support the CCM1 diagnosis.

Figure S5. Enriched KEGG pathways reaffirm the existence of complement and coagulation cascades caused by CCM1 deficiency in both Human and Mouse subjects.

A. The KEGG enrichment pathways for the Human enriched genome shows the complement and coagulation, including ECM components. While this was identified as a KEGG pathway, it

in the human CCM1 patient it supports the degraded binding suggested by the GO enrichment where binding proteins were found (Fig. S2A). **B. S**imilarly, the KEGG enrichment pathway analysis for the mouse Ccm1 subject highlighted the affected complement and coagulation cascades. This further supports our previous findings and strengthens the diagnosis of CCM1 deficiency.

S1A

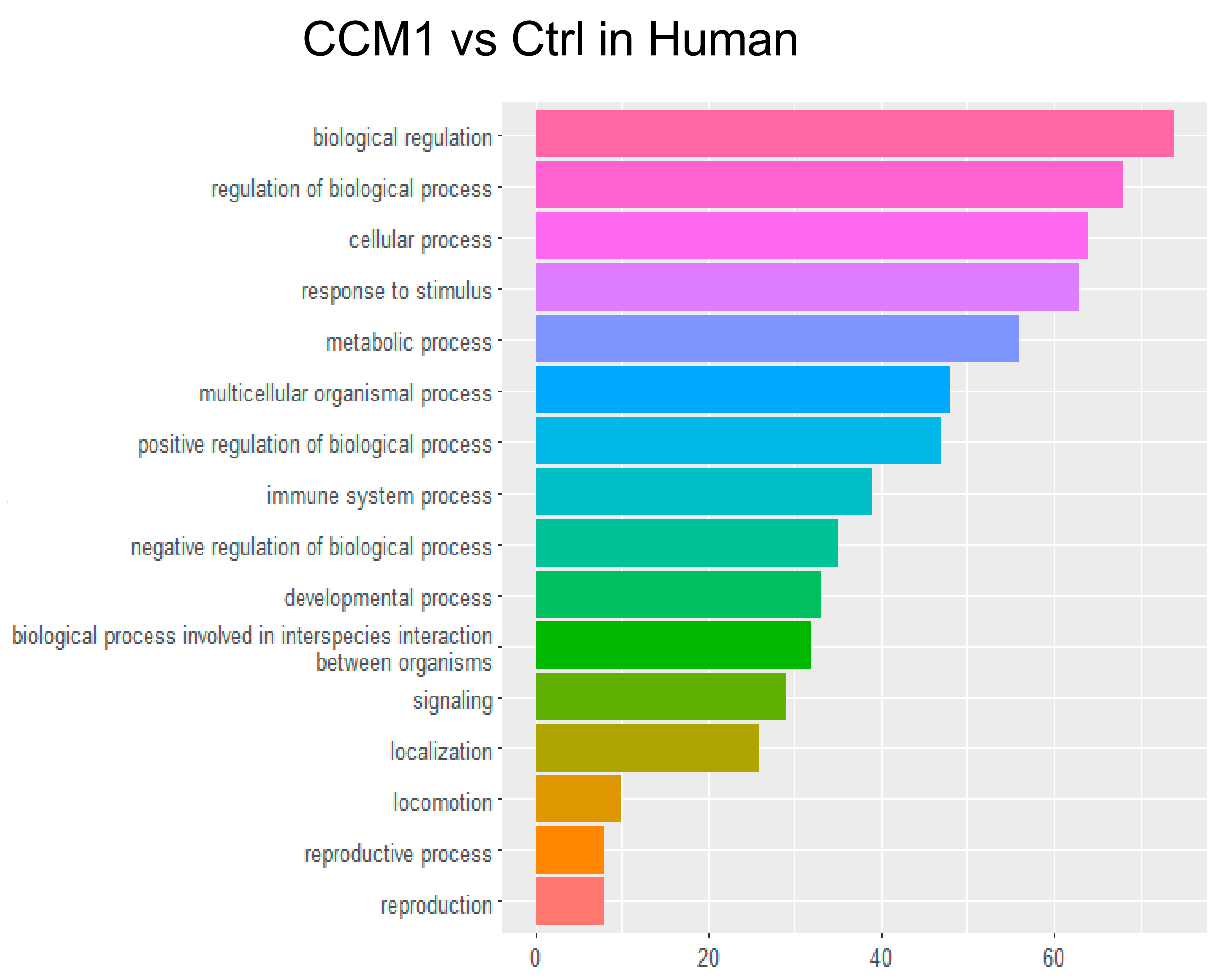

S1B

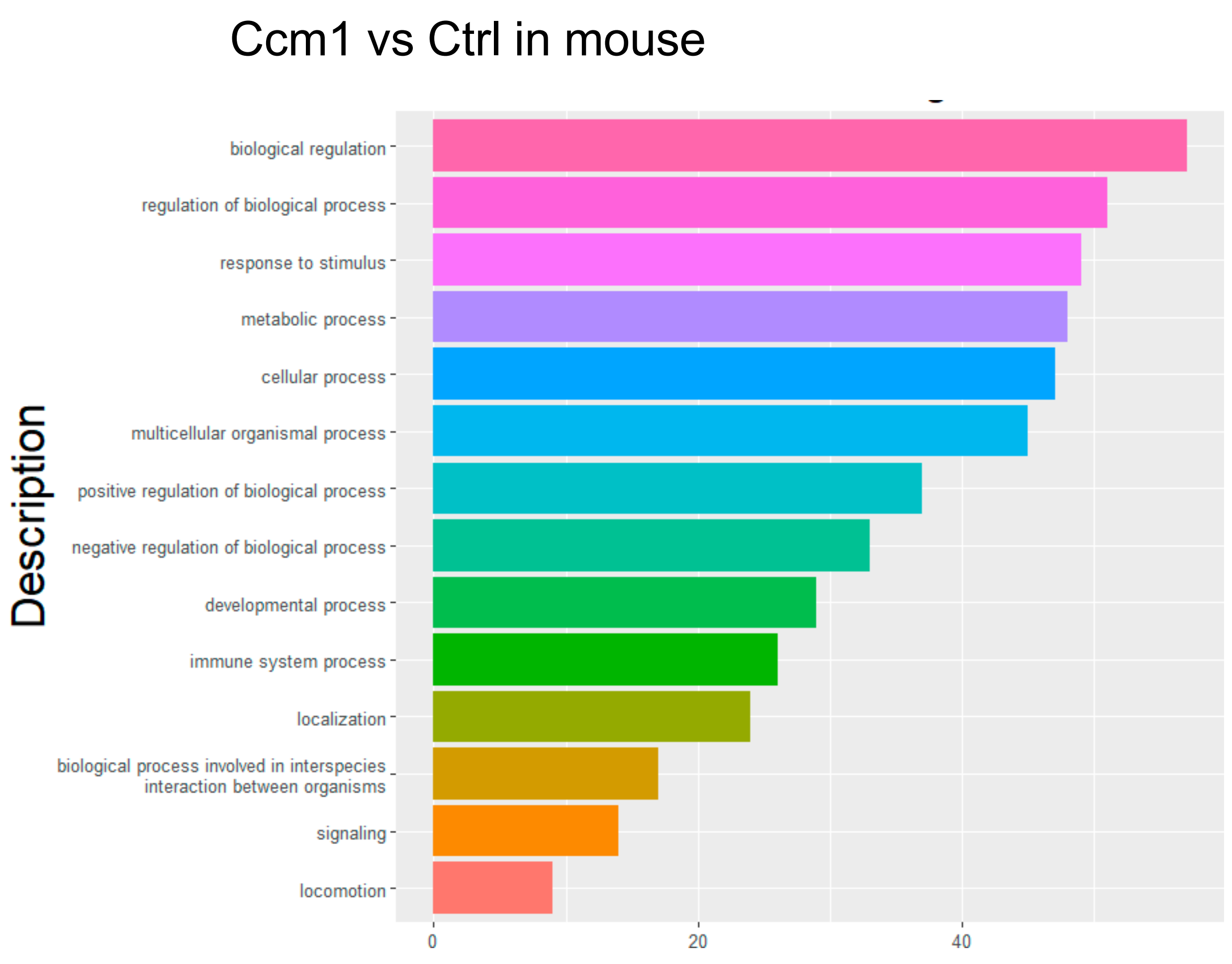

1. Group-GO Biological Process

S1C Human

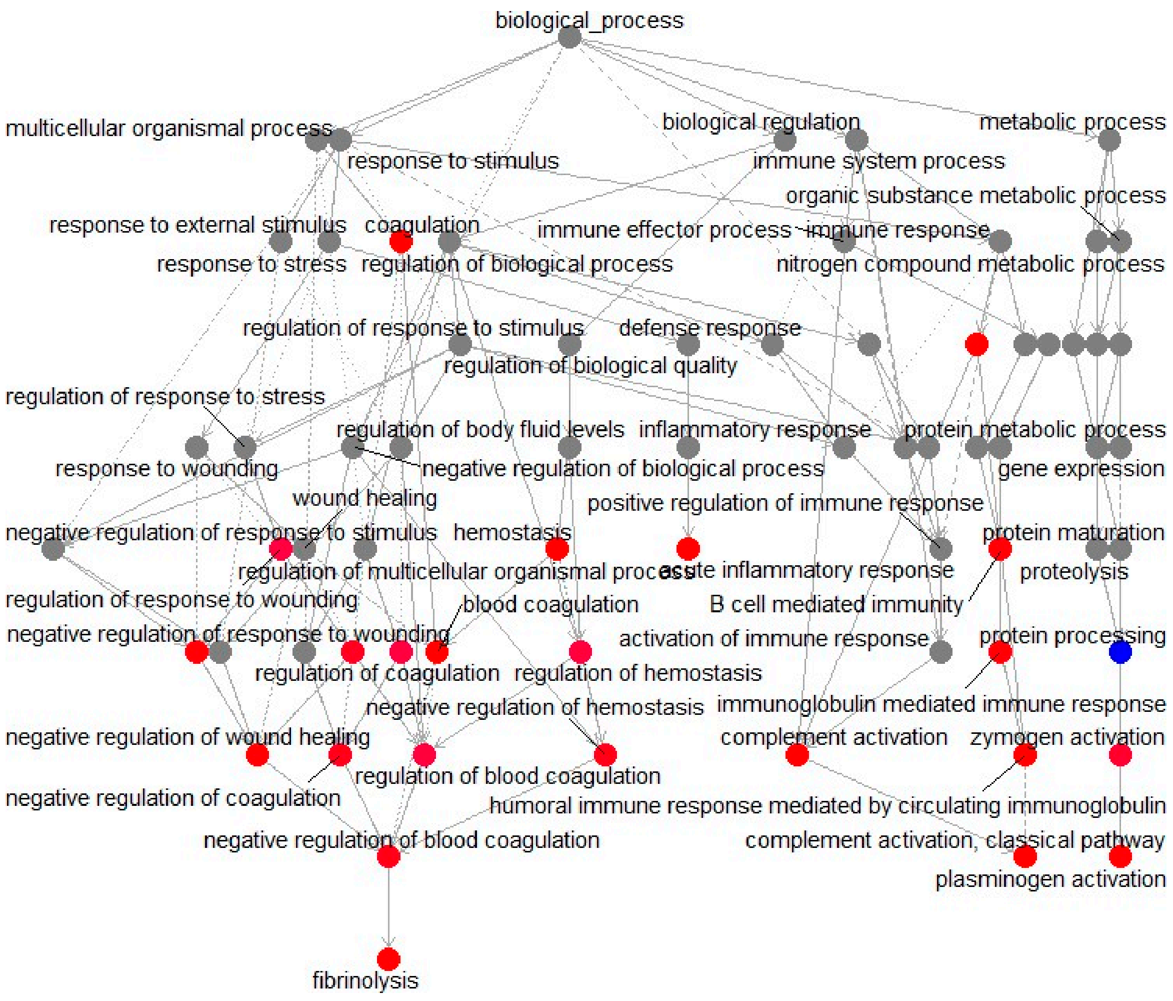

S1D Mouse

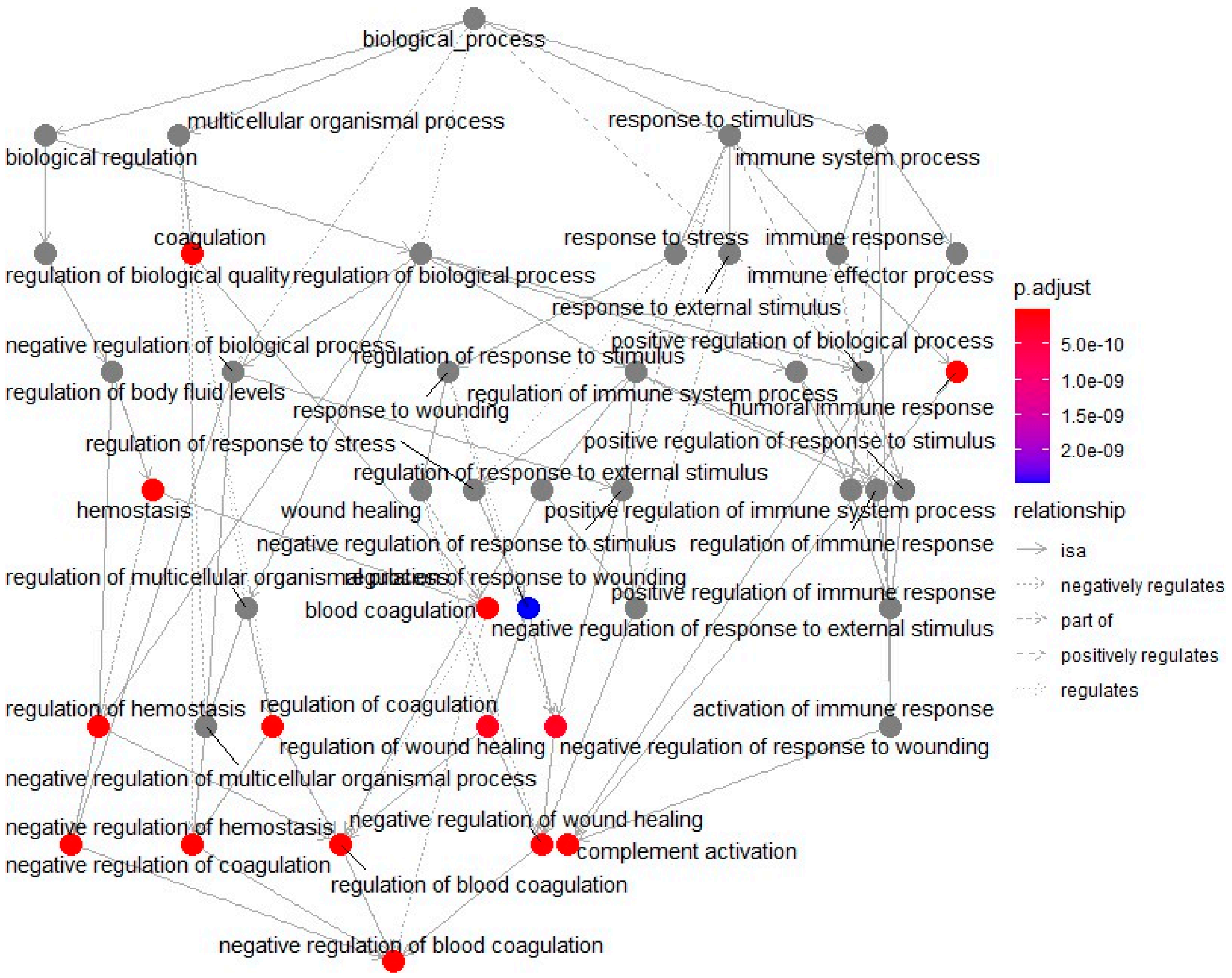

Enriched GO-Biological Process

S2A

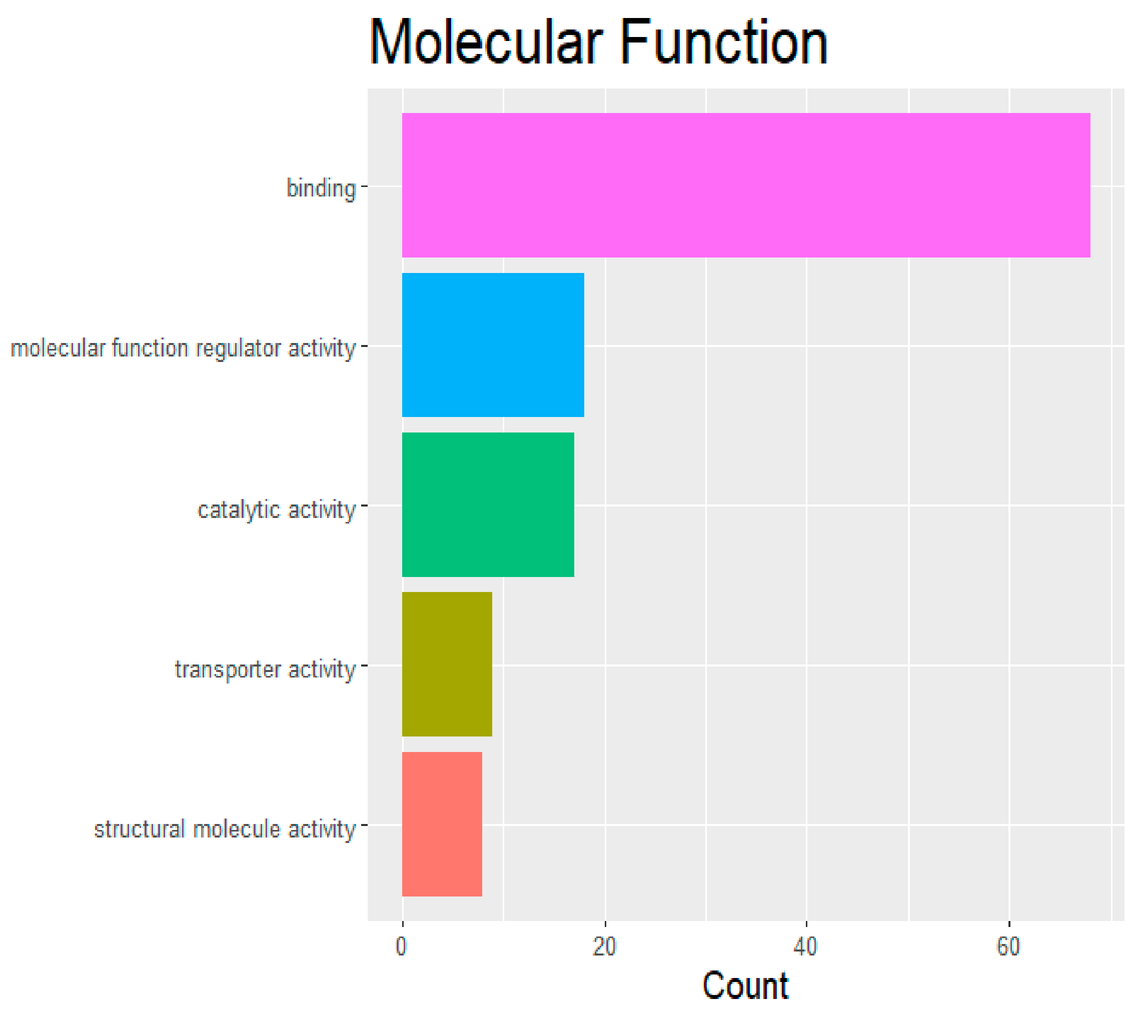
Molecular Function
binding
molecular function regulator activity
catalytic activity
transporter activity
structural molecule activity
0
20
40
60
Count

S2B.

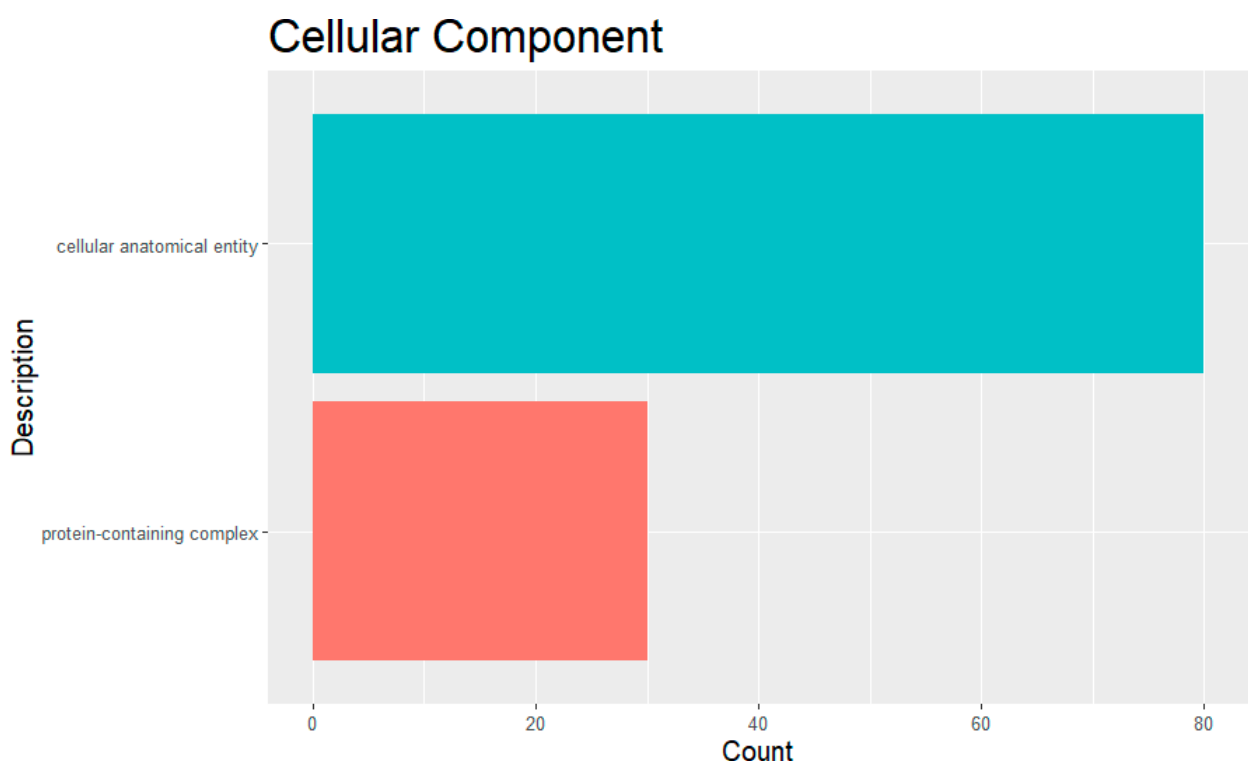
Cellular Component
Description
cellular anatomical entity
protein-containing complex
0
20
40
60
80
Count

S2C.

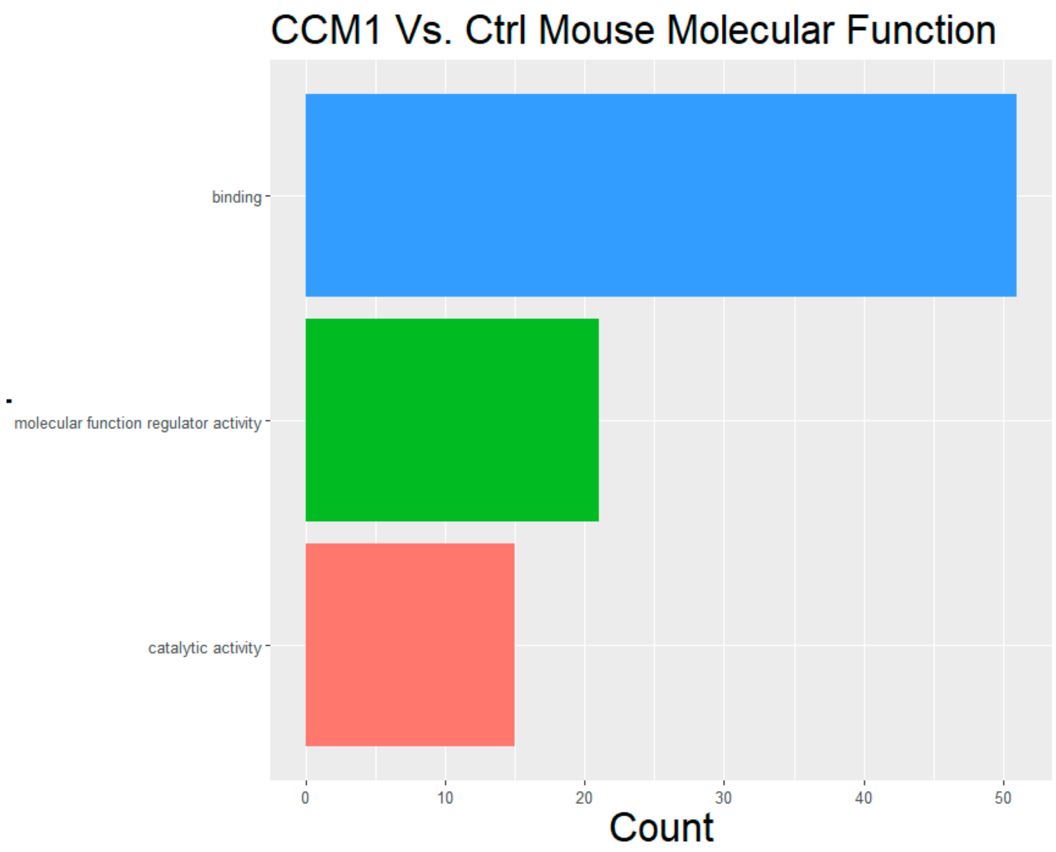
CCM1 Vs. Ctrl Mouse Molecular Function
binding
molecular function regulator activity
catalytic activity
0
10
20
30
40
50
Count

S2D.

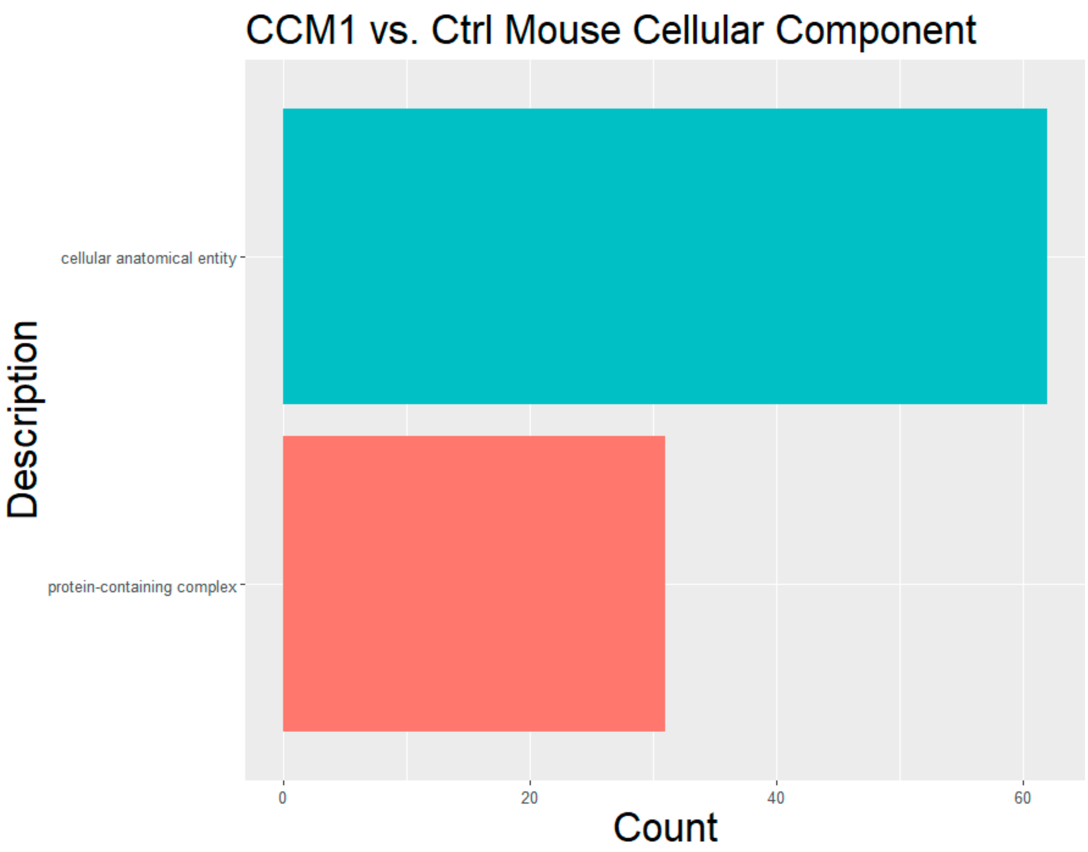
CCM1 vs. Ctrl Mouse Cellular Component
Description
cellular anatomical entity
protein-containing complex
0
20
40
60
Count

S2E. human

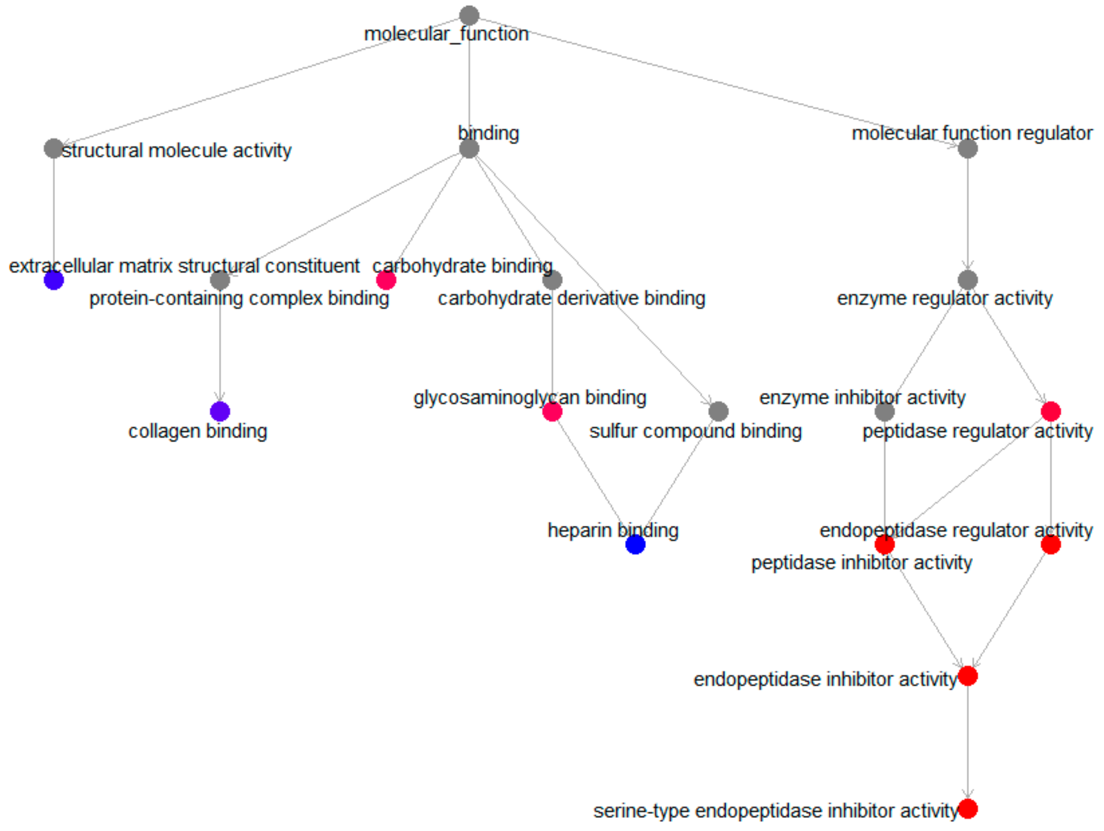

S2F.

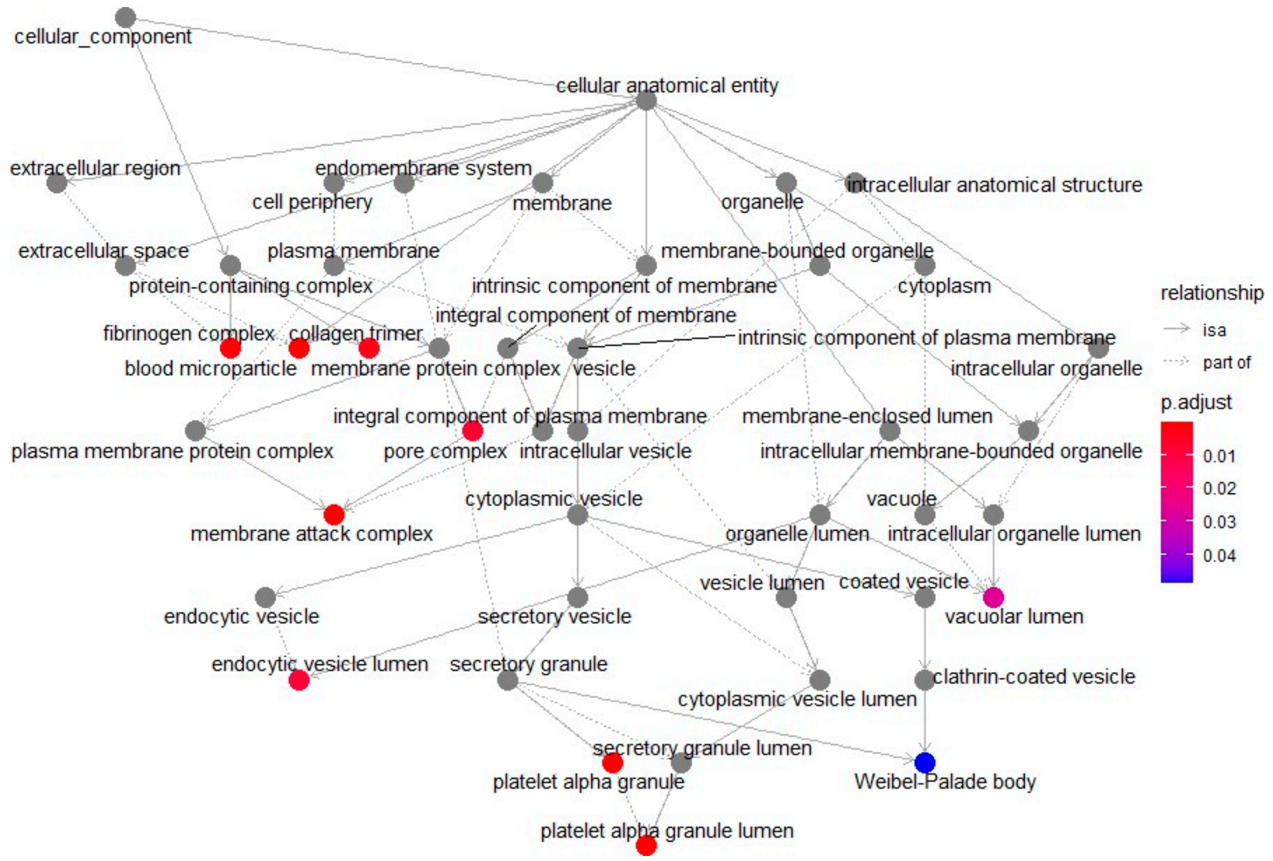

S2G. Mouse

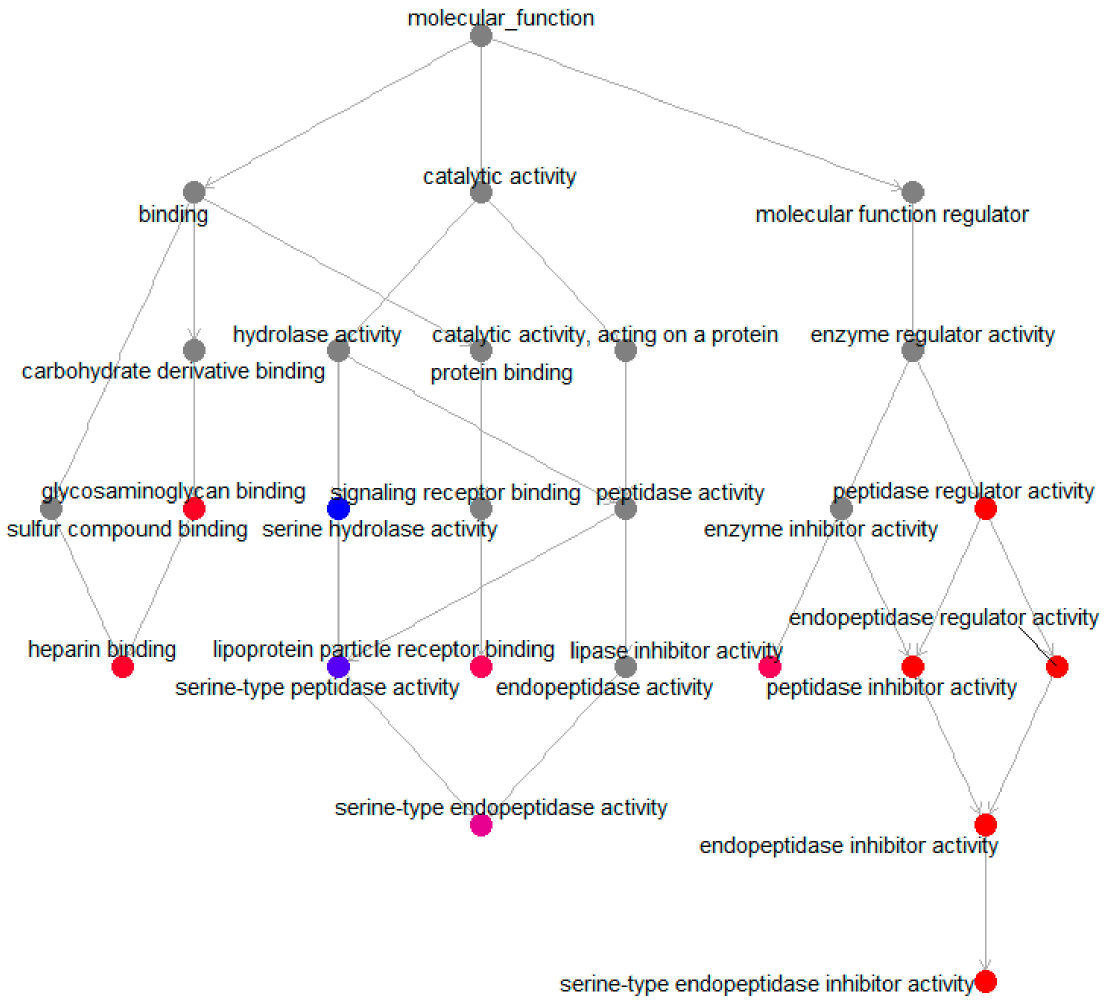

S2H.

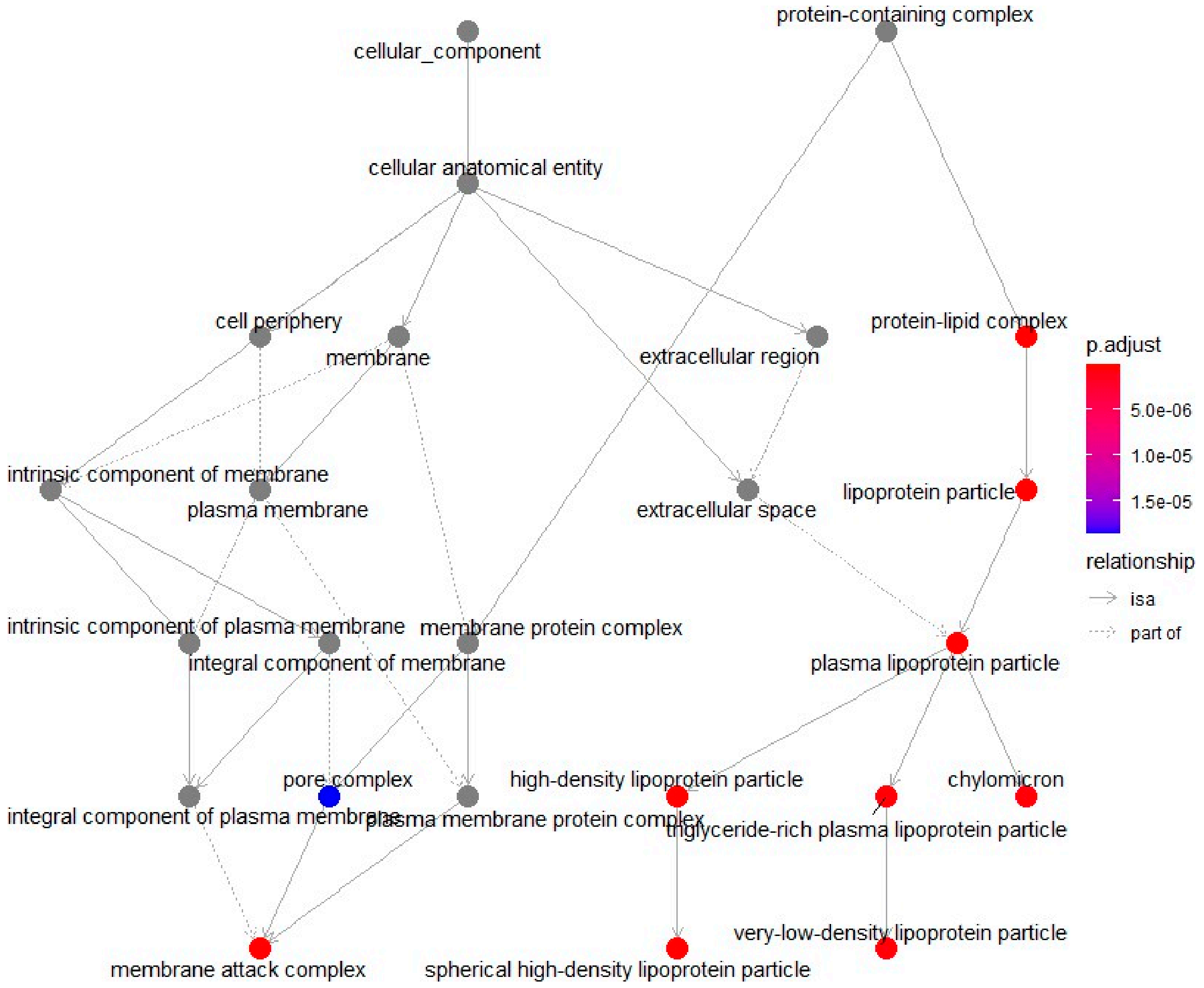

S3A.

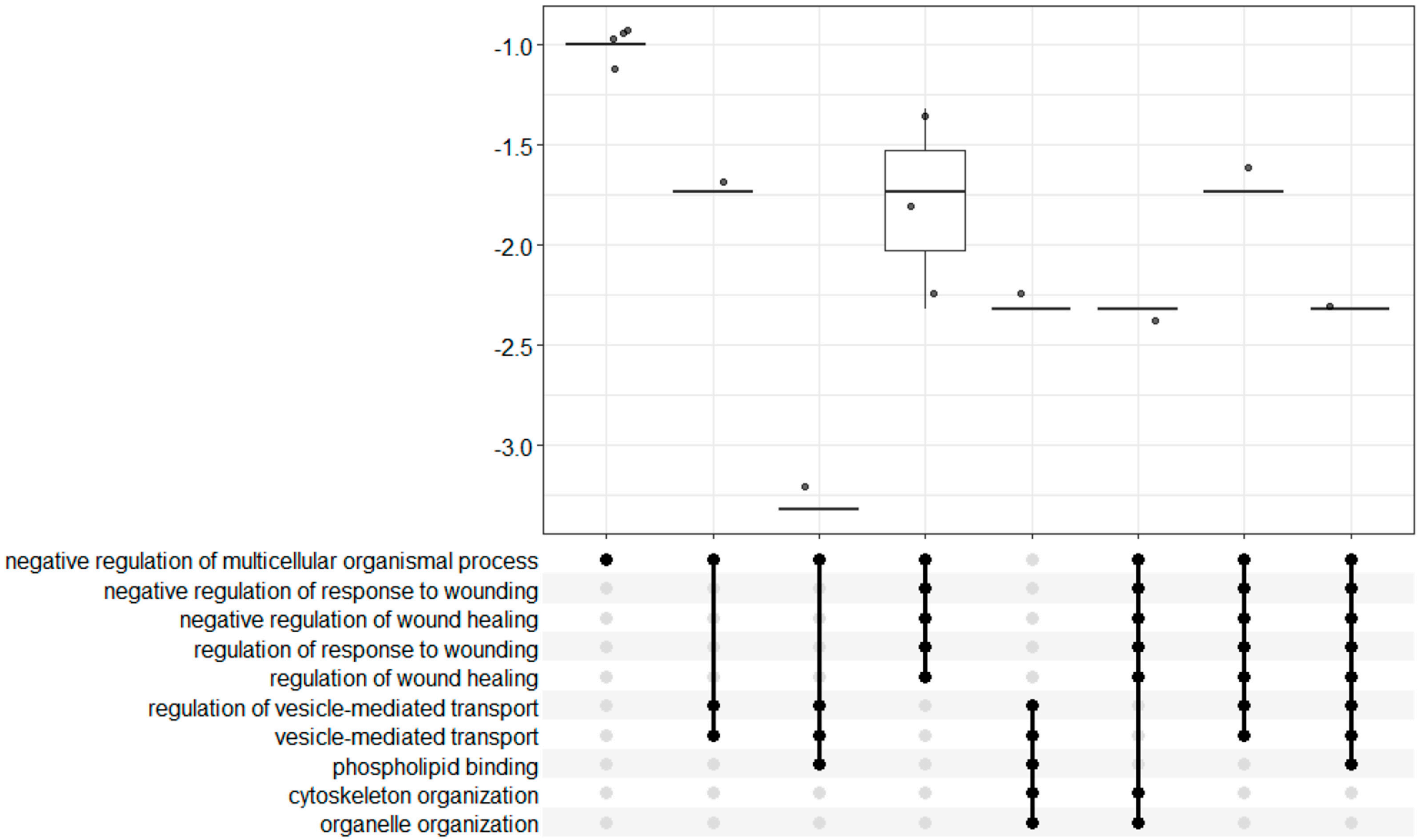
-1.0
-1.5
-2.0
-2.5
-3.0
negative regulation of multicellular organismal process
negative regulation of response to wounding
negative regulation of wound healing
regulation of response to wounding
regulation of wound healing
regulation of vesicle-mediated transport
vesicle-mediated transport
phospholipid binding
cytoskeleton organization
organelle organization

S3B.

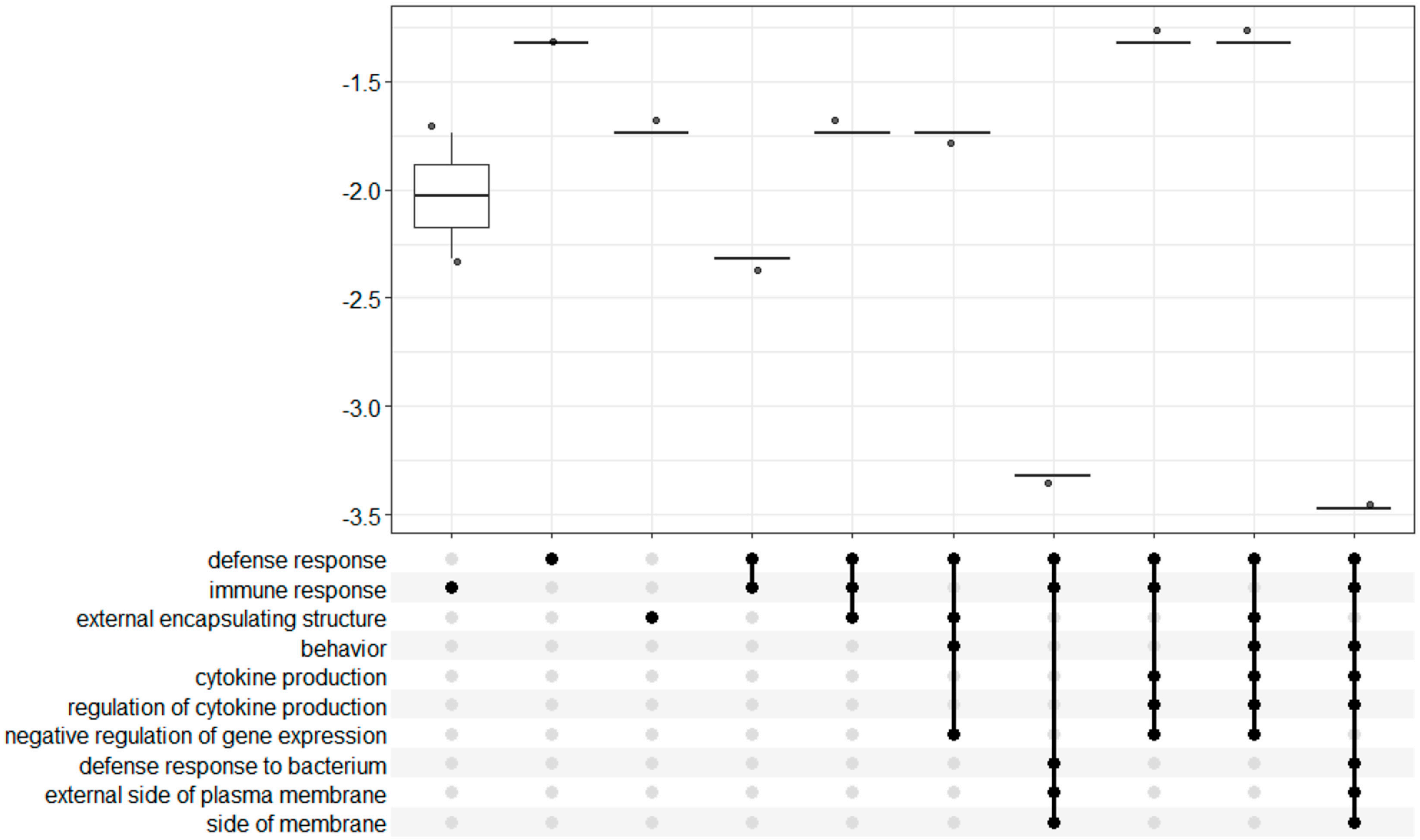

-1.5
-2.0
-2.5
-3.0
-3.5
defense response
immune response
external encapsulating structure
behavior
cytokine production
regulation of cytokine production
negative regulation of gene expression
defense response to bacterium
external side of plasma membrane
side of membrane

S4A.

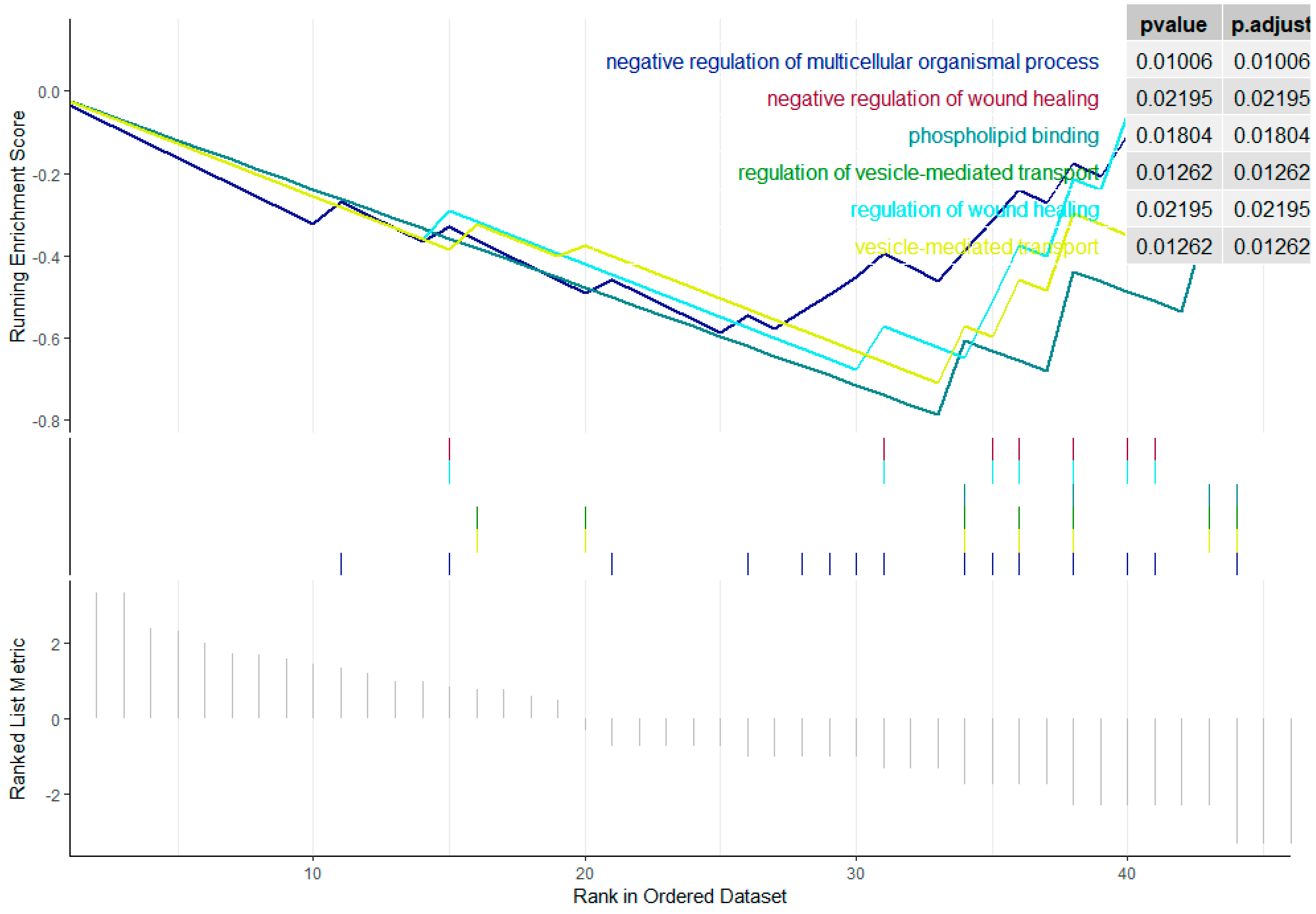

Running Enrichment Score
0.0
-0.2
-0.4
-0.6
-0.8
pvalue
p.adjust
negative regulation of multicellular organismal process
0.01006
0.01006
negative regulation of wound healing
0.02195
0.02195
phospholipid binding
0.01804
0.01804
regulation of vesicle-mediated transport
0.01262
0.01262
regulation of wound healing
0.02195
0.02195
vesicle-mediated transport
0.01262
0.01262
Ranked List Metric
2
0
-2
10
20
30
40
Rank in Ordered Dataset

S4B.

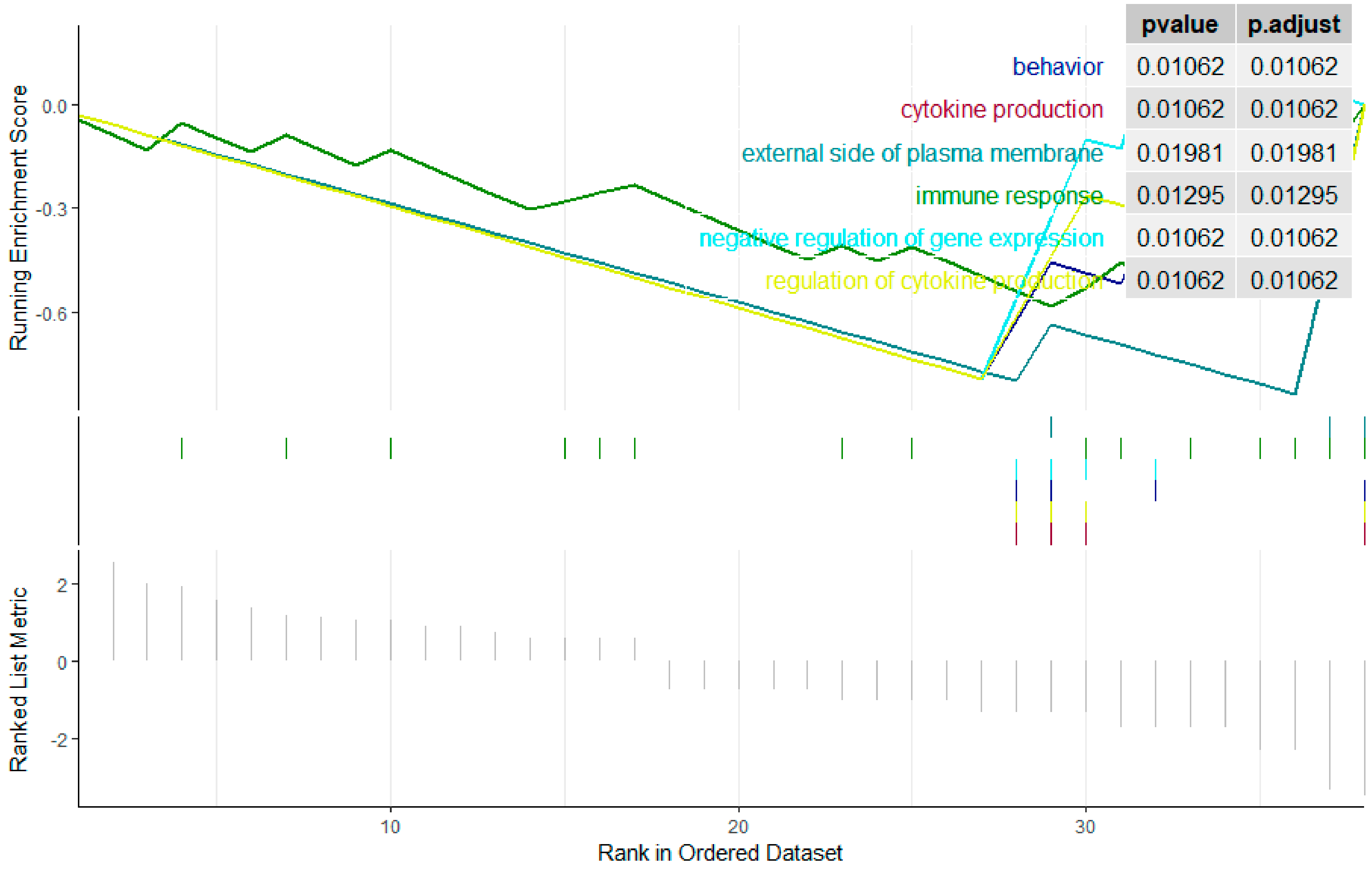

pvalue
p.adjust
behavior
0.01062
0.01062
cytokine production
0.01062
0.01062
external side of plasma membrane
0.01981
0.01981
immune response
0.01295
0.01295
negative regulation of gene expression
0.01062
0.01062
regulation of cytokine production
0.01062
0.01062
Running Enrichment Score
0.0
-0.3
-0.6
Ranked List Metric
2
0
-2
10
20
30
Rank in Ordered Dataset

S5A.
Human KEGG Enriched pathway

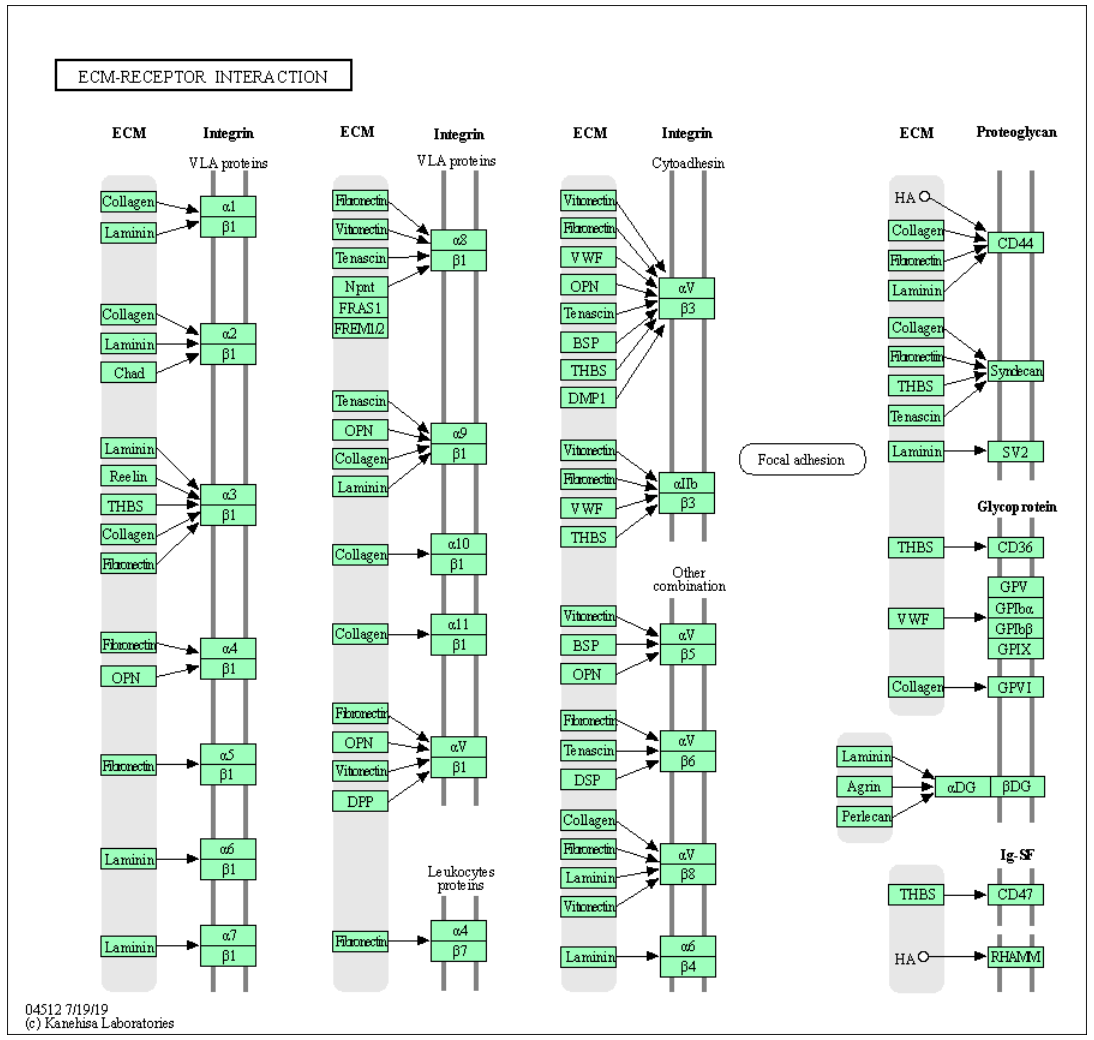

S5B.
Mouse KEGG Enriched Pathway

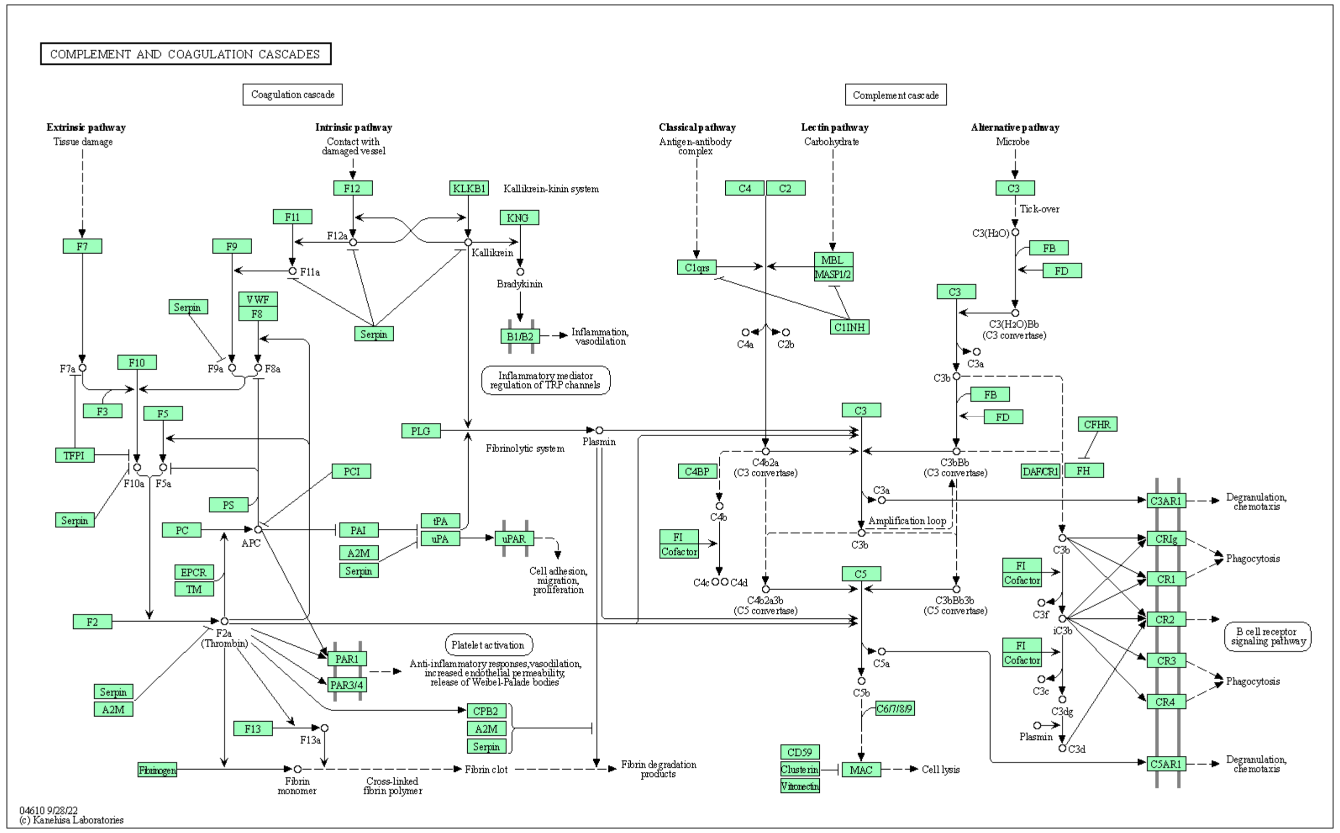

Supplement: Supplementary file 1 [file ijms-25-04740-s001.zip › ijms-2925006-Supplementary Material.pdf]
